# Reducing Afterpulsing in InGaAs(P) Single-Photon Detectors with Hybrid Quenching

**DOI:** 10.3390/s20164384

**Published:** 2020-08-06

**Authors:** Junliang Liu, Yining Xu, Zheng Wang, Yongfu Li, Yi Gu, Zhaojun Liu, Xian Zhao

**Affiliations:** 1 School of Information Science and Engineering, Shandong University, 72 Binhai Road, Qingdao 266237, China; junliang_liu@sdu.edu.cn (J.L.); 18251300116@163.com (Z.W.); zhaojunliu@sdu.edu.cn (Z.L.); 2 Key Laboratory of Education Ministry for Laser and Infrared System Integration Technology, Shandong University, 72 Binhai Road, Qingdao 266237, China; 201812563@mail.sdu.edu.cn (Y.X.); zhaoxian@sdu.edu.cn (X.Z.); 3 Center for Optics Research and Engineering, Shandong University, 72 Binhai Road, Qingdao 266237, China; 4 Key Laboratory of Infrared Detection and Imaging Technology, Shanghai Institute of Technical Physics, Chinese Academy of Sciences, 500 Yutian Road, Shanghai 200083, China; guyi@mail.sitp.ac.cn

**Keywords:** single-photon detectors, single-photon avalanche diodes, avalanche photodiodes, quenching circuits

## Abstract

High detection efficiency appears to be associated with a high afterpulse probability for InP-based single-photon avalanche diodes. In this paper, we present a new hybrid quenching technique that combines the advantages of both fast active quenching and high-frequency gated-passive quenching, with the aim of suppressing higher-order afterpulsing effects. Our results showed that the hybrid quenching method contributed to a 10% to 85% reduction of afterpulses with a gate-free detection efficiency of 4% to 10% at 1.06 μm, with 40 ns dead time, compared with the counter-based hold-off method. With the improvement of the afterpulsing performance of high-frequency gated single-photon detectors, especially at relatively high average detection efficiencies with wide gate widths, the proposed method enables their use as high-performance free-running detectors.

## 1. Introduction

InGaAs/InP single-photon avalanche diodes (SPADs) have emerged with the need for near-infrared single-photon detection by applications, such as quantum key distribution (QKD) [1] and light detection and ranging (LiDAR) [2]. Compared to superconducting single-photon detectors (SPDs), which require cryogenic cooling to reach a superconducting state [3], SPADs do not operate at extremely low temperatures and are easy to integrate into systems. However, compared to other detectors, such as superconducting SPDs, silicon-based SPADs, or photo multipliers, the afterpulsing effects in InP-based SPADs induce more false counts. Afterpulsing refers to a second or more of spurious avalanche pulses ignited by the spontaneous release of previous avalanche carriers trapped by defects, and the probability of having an afterpulse soars as the detection efficiency increases [4].

Striking a balance between afterpulse probability and detection efficiency has been a long-lasting problem for InGaAs SPADs. For a given SPAD, there are typically two approaches to restrain the afterpulsing effects without sacrificing the detection efficiency. One is to hold the SPAD off for a period of time (called the “hold-off time” or “dead time”) after an avalanche event. This method is present in almost all free-running InP-based detectors. While the maximum detection efficiency is typically around 25% [5,6,7], or even reaches 35% [8,9] for free-running detectors, the afterpulse probability can be as high as 1000% at a low hold-off time of 100 ns and 20% detection efficiency, forcing the user to choose a longer hold-off time. As trapped carriers typically exhaust after tens of microseconds at 223 K, a 20 μs hold-off time can sufficiently keep the afterpulse probability lower than 1% at a detection efficiency of up to 20% [5]. However, such a long hold-off time apparently lowers the maximum count rate, and makes a free-running detector unworthy of its name for it is often “dead”. Therefore, balanced performance is possibly favored. For example, the detection efficiency should be kept below 10% for a hold-off time of 100 ns [4]; otherwise, the hold-off time should typically be set to 1 μs or longer for a detection efficiency above 15% [4,8].

The second approach is to quench the SPAD more quickly so as to have a smaller amount of avalanche carriers without sacrificing the count rate [10]. As is well known, SPADs have to work under the condition called “Geiger mode”, i.e., the bias voltage is higher than the breakdown voltage, to obtain a sufficient magnification factor for a single photon absorbed to generate a detectable avalanche pulse. Once the avalanche process is triggered, it can be stopped only if the bias voltage falls below the breakdown voltage, which “quenching” represents.

Among ordinary quenching techniques, gated-passive quenching is quite popular for high-count-rate applications [11]. A periodical gating signal is used in this technique to enable the SPAD to generate avalanche pulses by over-biasing it within the signal peaks and disable the SPAD or quench the avalanche by under-biasing it outside the peaks. If there is an avalanche, the bias voltage is quenched within the gate-off period. This quenching technique is still a “passive” one, as no active feedback is involved. Compared with other passive or active quenching techniques, the avalanche pulses generated with this technique are much shorter because the effective gate width is only hundreds of picoseconds [12]. As a result, afterpulsing is typically suppressed to a one-digit percent value [13,14,15].

As the dead time becomes nanosecond-scale, the count rate reaches hundreds of megahertz [14], compared with state-of-the-art free-running InP-based detectors having a dead time of above 50 ns [8]. In addition, the peak detection efficiency reaches 50% at 251 K, 1310 nm [16], and 55% at 293 K, 1550 nm [15]. However, the high detection efficiency applies only when the majority of the incident photons are within the short gate-on period. When used as a free-running detector, where the light source does not have to be synchronized with the gate, the average detection efficiency falls to approximately 20% of the peak, which is typically 4% to 7%, depending on the duty cycle of the gating signal [14]. A smaller duty cycle contributes to a lower afterpulse probability but a lower average detection efficiency as well. This technique can still be used in free-running applications, such as 3D LiDAR [17], which require low afterpulse probability and low dead time, with a relatively low detection efficiency.

In this paper, a hybrid quenching method is presented, which combines gated-passive quenching and our fast active-quenching technique with the aim of reducing high-order afterpulsing effects at high detection efficiency in single-photon detectors based on InGaAs(P)/InP SPADs. SPDs with two models of InGaAs(P) SPADs of different targeting wavelengths and active areas were built with the proposed hybrid-quenching circuit, and were optimized for high average detection efficiency and low afterpulse probability in free-running applications. The performance of hybrid quenching and the proposed SPDs were evaluated, in particular for the afterpulsing probabilities, at various conditions by comparing the results of different methods.

## 2. Method

The core of the detector is the SPAD driven by the hybrid quenching circuit. The circuit was designed with a basic concept of adding active quenching to a high-frequency gated-passive quenching circuit. The avalanche can be quenched by high-frequency gated-passive quenching within a nanosecond, and hence the number of trapper avalanche carriers is significantly lower than with other techniques. However, the following gates are reopened every next nanosecond, and the afterpulses are likely to occur in those gates especially when the excess bias is high. These afterpulses will trigger more afterpulses later, and eventually contribute to a large number of afterpulses. This phenomenon is usually called the higher-order afterpulsing effect [18]. When active quenching is added with high-frequency gated-passive quenching, it can maintain the excess bias at a value below break down voltage for a short period of time, e.g., the first tens of nanoseconds, which physically closes the following tens of gates after an avalanche event and prevents the release of afterpulses within these gates.

Sophisticated design is required to apply a DC bias, high-frequency gating signal, and active-quenching signal to the SPAD, along with the avalanche current pick-up circuit. On the one hand, one cannot simply turn off the high-frequency gating signal after an avalanche to achieve this goal, as the DC bias voltage can be above the breakdown voltage, and doing this would leave the SPAD with merely passive quenching. On the other hand, both the active-quenching signal and high-frequency gating signal can feed through the junction capacitor of the SPAD, and such transient responses become noise in the output signal of the SPAD. Many techniques have been developed to cancel this kind of noise. The tunable self-differencing method [19] is preferred for high-frequency gating due to its excellent attenuation of noise as well as its low distortion of the avalanche pulse, and hence outstanding performance was achieved [15]. Low-pass filtering is another popular option for noise suppression in high-frequency gating schemes [20], and was developed to be compact and robust [12]. However, these methods alone are not applicable for noise induced by the non-periodical active-quenching signal.

Another favorable method is capacitor balancing [21], including diode balancing [22] and the dummy-SPAD balancing [14] technique. This method is suitable for a wide frequency range and a variety of designs from low-frequency gating [23] to variable high-frequency gating [21], and even for free-running SPDs [4]. In particular, when the capacitor-balancing method is used with high-frequency gating, low-pass filters are often applied as a supplement to remove the residual higher-order component of the noise [14,21].

**Figure 1:**
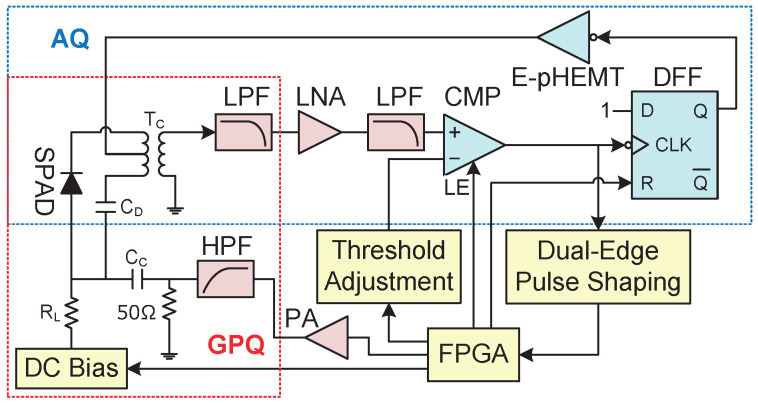
Schematic of the proposed quenching circuit combining gated-passive and active quenching techniques. AQ: active quenching. CMP: comparator. DFF: D-type flip-flop register. E-pHEMT: enhancement-mode pseudomorphic high electron mobility transistor. FPGA: field-programmable gate array. GPQ: gate-passive quenching. HPF: high-pass filter. LE: latch enable. LNA: low-noise amplifier. LPF: low-pass filter. PA: power amplifier. SPAD: single-photon avalanche diode.

We chose the latter method for the gated-passive quenching part of our design, as shown in Figure 1. The high-frequency gating signal is generated by the multi-gigabit transmitter of the field-programmable grid array (FPGA, Xilinx XC7A35T-2CSG325I). The data rate are set to 6.25 Gbps, and the data pattern is “0011” to obtain a clock rate of 1.5625 GHz. The gating signal is amplified by a two-stage power amplifier (PA) with a total gain of 34 dB and a bandwidth of 6 GHz into approximately 14 $V_{\mathrm{pp}}$, and this signal passes through a high-pass filter (HPF) of 1.25 GHz to eliminate the wide-band noise coming out of the transmitter of the FPGA.

Both the gating signal and the DC bias are applied on the anode of the SPAD through a coupling capacitor $C_{C}$ and a current-limiting resistor $R_{L}$, respectively. For the avalanche current pick-up and noise canceling, the cathode and the dummy capacitor $C_{D}$ are connected to the primary winding of a wide-band radio-frequency (RF) transformer $T_{C}$, and hence only the avalanche current is transformed to the secondary side. The avalanche signal with noise residue is filtered by two 900-MHz low-pass filters, and is amplified by 31 dB using a wide-band low-noise RF amplifier before entering the comparator. To minimize the delay of the signal conditioning chain, the low-pass filters are low temperature co-fired ceramic (LTCC) ones with a small size of 2.0 mm by 1.2 mm, and are installed on the printed circuit board along with the amplifier and the transformer.

The active-quenching part is shown in Figure 1. A D-type flip-flop register (DFF) is used for holding off the SPAD once triggered by the comparator. The output of the DFF is amplified by an enhancement-mode pseudomorphic high electron mobility transistor (E-pHEMT) to quench the SPAD. The hold-off time is controlled by the FPGA, and the reset of the SPAD is performed by resetting the DFF. The active-quenching signal coming out of the E-pHEMT is transmitted to the cathode of the SPAD through the center tap of the primary winding of the RF transformer. The active-quenching signal passes through both the junction capacitor of the SPAD and the dummy capacitor, with two identical transient currents flowing toward opposite directions in the winding. Thus, ideally there is no noise current flowing through the secondary winding, and the noise is removed.

However, as the transformer is not ideal, and noise residue induced by the active-quenching signal can pass through LPFs and the LNA, becoming amplified and distorted but not filtered out. As the noise lasts only several nanoseconds, the latch function of the comparator is enabled during reset to lock itself until the noise residue settles down, to prevent false triggering without increasing the discrimination threshold.

## 3. Experimental Setup

**Figure 2:**
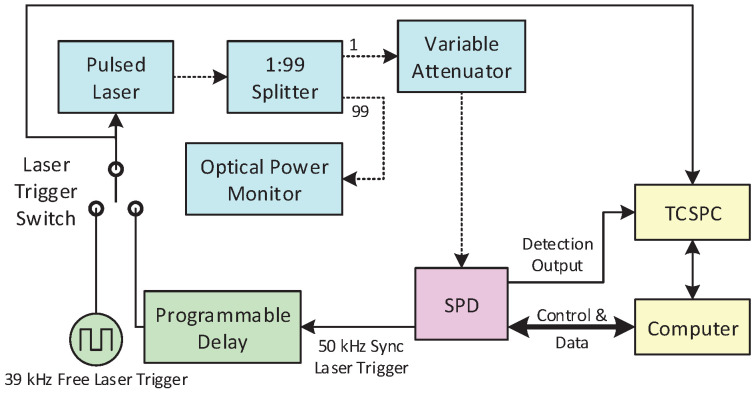
The experimental setup for the performance evaluation of the proposed single-photon detector (SPD). TCSPC: time-correlated single-photon counter. The dashed lines represent single-mode optical fibers, and the solid lines represent coaxial electrical connections.

An experimental system was built for the characterization of the SPD, shown in Figure 2. Two types of InP-based SPADs were tested: InGaAs SPAD (PGA-300, Princeton Lightwave) with an active area of 25 μm diameter, suitable for 0.95 to 1.65 μm photon detection; and InGaAsP SPAD (PGA-384, Princeton Lightwave, Cranbury, NJ, USA) with a 80 μm diameter, suitable for 0.95 to 1.1 μm photon detection. The PGA-series devices are commercially available InP-based SPADs specifically designed for single-photon detection. They have demonstrated good overall performance; however, they both have the typical problem of high afterpulsing and a long afterpulsing lifetime, which poses a high demand on the quenching electronics. PGA-300 and PGA-384 share similar structures and multiplication layers; however, they have different active areas and absorbing layer materials. The differences make them good examples for a comparative performance evaluation of the proposed quenching method.

The photon source used in the experiments was a pulsed laser source (PDL 800-B, picoQuant, Berlin, Germany) with interchangeable laser diode heads of 1.06 and 1.55 μm. As the SPD is proposed for both gated and free-running applications, the trigger source of the laser has two options. One is 50 kHz synchronized with the 1.5625 GHz gating signal, which results in one laser pulse every 3125th gate. A 10-ps precision programmable delay module (SY89295U, Microchip, Chandler, AZ, USA) was inserted between the laser and the trigger source to adjust the time of arrival of the photon in the gate. The other laser trigger source was 39.0625 kHz from an independent oscillator, unsynchronized with the gating signal. The laser was attenuated to 1 photon per pulse on average for afterpulsing measurements, and was attenuated to 0.1 photon per pulse on average for other tests if needed. A dual-channel time-to-digital converter with 36 ps resolution and 3.75 ns dead time and two gated counters with 5 ns dead time were all embedded in the FPGA for performance evaluation, except for the jitter, which was measured with a high-resolution time-to-digital converter (quTAG, quTools, München, Germany) set to 2 ps bin resolution.

The performance of the proposed SPD can be characterized by the gate width, photon detection efficiency (PDE, η), dark count rate (DCR, $C_{d}$), afterpulse probability, and timing jitter. As the SPD aims at free-running applications, the dark count rate in this paper is represented by the plain number of raw dark counts per second instead of the dark count probability per gate. Considering the condition of gate-free detection, the PDE can be calculated here simply by:

(1) $$\eta =\frac{C_{i}-\tau_{w}f_{L}C_{d}}{\mu f_{L}}$$

where $C_{d}$ is the raw count rate without illumination, $C_{i}$ is the count rate within the illuminated window in the time histogram obtained with time-correlated single-photon counter (TCSPC), $\tau_{w}$ is the width of the illuminated time window, $f_{L}$ is the repetition rate of the pulsed laser, and μ is the average number of photons per pulse.

Afterpulsing is evaluated through the total afterpulse probability and the time distribution of the afterpulses, i.e., the afterpulse probability density. The time distribution of the afterpulse probability is represented by the afterpulse probability per unit time (e.g., 1 ns) after a certain time *t*, and can be calculated from the time histogram of count events obtained by TCSPC, with the following equation:

(2) $$P_{ap}\left(t\right)=\frac{1}{\tau_{b}}\times \frac{C\left(t\right)-\tau_{b}f_{L}C_{d}}{C_{i}-\tau_{w}f_{L}C_{d}},$$

where $C\left(t\right)$ is the count rate within the time bin at the time *t* after the photon detection, and $\tau_{b}$ is the bin width of the histogram. The total afterpulse probability was then calculated through integrating the afterpulse probability density.

In order to assess the performance of hybrid quenching, the measurements were implemented under high-frequency gated operation with additional hold-off time applied using three methods separately: a minimum hold-off time limited by hardware, a counter-based hold-off time by screening count events, and true hold-off times by active quenching. Post-processing dead time using software has been proven effective [15], and is equivalent to count-based dead time in our experiments.

## 4. Results and Discussion

**Figure 3:**
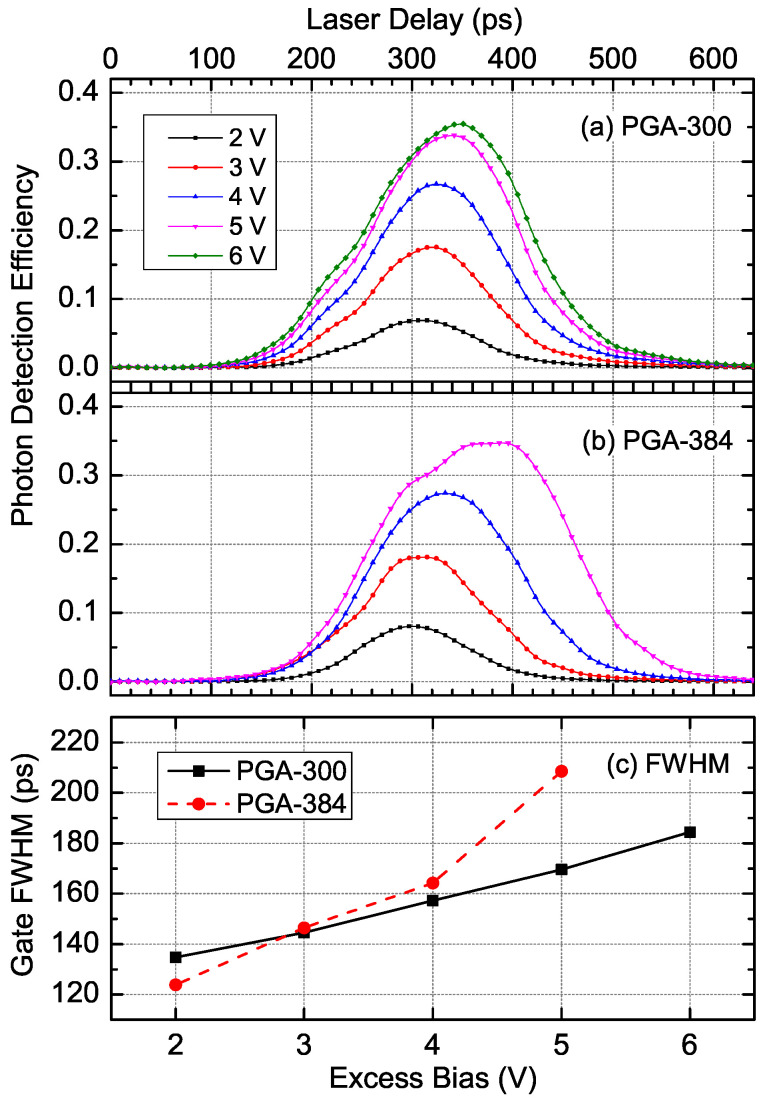
The photon detection efficiency (PDE) of (**a**) PGA-300 and (**b**) PGA-384 within the gate at various excess bias voltages, and (**c**) the full-width at half-magnitude (FWHM) of the effective gate, all measured at 1.06 μm wavelength, 223 K.

For a high-frequency gated detector to be used both as a gated detector and a free-running one, the gate width first needs to be characterized. The shape of effective gates under different excess bias for PGA-300 and PGA-384 are shown in Figure 3a,b, respectively, and the effective gate widths were calculated accordingly, shown in Figure 3c. Unlike the results in other studies, the gate widths of the proposed detector were generally large, up to 210 ps, as shown in Figure 3c.

Typically, the gate width is expected to be as narrow as possible for synchronized gated detection in order to have a lower afterpulse probability. Sinusoidal or harmonics-synthesized [16] pulsed gating signals with large amplitude are often used for this reason. For example, an afterpulse probability of as low as 1.5% was reported with a gate width up to 100 ps, which is only 13% of the 1.3 GHz gate [14]. However, as one of the goals of the research was to gain higher detection efficiency for free-running applications, a square-wave gating signal was selected on purpose instead of a sine wave to have a larger effective gate width of around 200 ps, which is 31% of the gate. Under synchronous detection circumstance, a wider gate provides larger tolerance to the fluctuation of the timing of incident photons, which could be introduced by the fiber in the QKD system, for example.

In addition, the integration of the active-quenching technique into gated-passive quenching extends the usable excess bias range of the SPD to achieve even higher detection efficiency, at the cost of a longer dead time. For instance, the results at 5 V or above in Figure 3a–c were all obtained with the help of active quenching at a 1 μs hold-off time; otherwise the false counts (including the dark counts and afterpulses) would have gone out of control for the current setup. However, the false count rate will dramatically increase if the excess bias is tuned higher, without an obvious increase of detection efficiency. Since there is always a dead time, an extremely large number of false counts could dominate most of the detection gates, and inhibit the detection of photons by forcing the SPAD to be “dead” too often. Besides, compound semiconductor transistors, such as GaAs E-pHEMT, have a lower break-down voltage than silicon ones, which sets the upper limit of the excess bias. Therefore, the maximum detection efficiency is limited both by the false count rate and the circuit.

Both PGA-300 and PGA-384 were operable under the 1.56 GHz gating signal; however, the effectiveness of the gates was different, as can be seen from Figure 3a,b. Despite the similar peak detection efficiency of approximately 35%, the maximum operable excess bias of PGA-384 was lower. Here, the description “maximum operable” refers to the value when the peak detection efficiency keeps rising while the dark count rate does not soar. The tendency of broadening of the gate was different for the two devices. While PGA-300 had an ordinary “Gaussian” appearance (Figure 3a), PGA-384 exhibited an extension of the detection closer to the tail of the gate with a flat-top detection peak at 5 V excess bias (Figure 3b). The full-width at half-magnitude (FWHM) of the effective gate rose more rapidly for PGA-384 with the increase of the excess bias (Figure 3c). These results indicate that the gate was less effective for PGA-384 due to its larger effective area, and the afterpulse probability was then higher than that of PGA-300.

The following results were all obtained using incident photons unlocked to the gating signal, in order to demonstrate the performance of the SPD in free-running mode. For PGA-384, the excess bias of 2 to 5 V corresponded to a gate-free PDE of 3.5% to 13.4% at 1.06 μm, and for PGA-300, 2 to 6 V corresponded to a gate-free PDE of 2% to 13% at 1.06 μm, or 1.4% to 9.3% at 1.55 μm.

**Figure 4:**
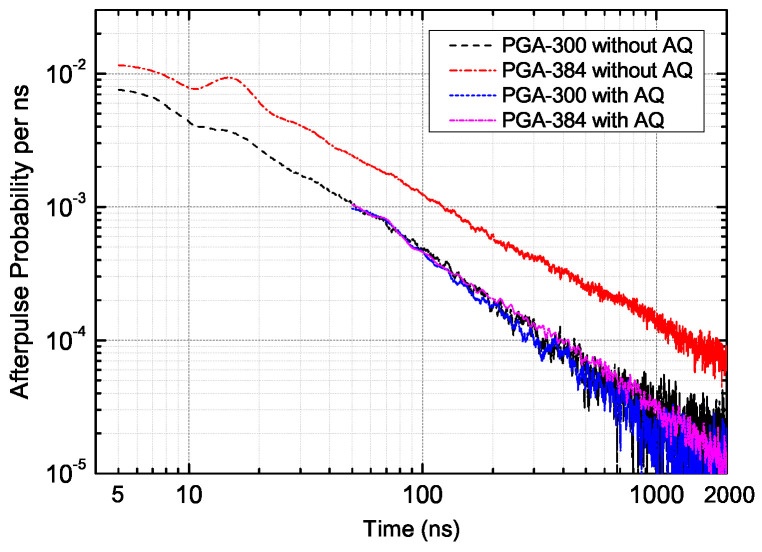
Time distribution of the afterpulse probability of PGA-300 and PGA-384, with and without active quenching (AQ), measured at PDE = 8%, λ=1.06μm, 223 K.

Finding suitable conditions and the optimization of the hold-off time with active quenching requires an investigation of the time distribution of the afterpulse probability. For instance, the afterpulse probabilities per nanosecond as a function of time for PGA-300 and PGA-384, both at PDE = 8%, 1.06 μm, are shown in Figure 4. The small bumps around 13 ns are regarded as a result of the pile-up effect due to the dead time of the detector and measurement hardware, and also as a sign of the high afterpulse probability.

The drawback of having a larger detection area is clear: the afterpulse probability without active quenching for PGA-384 was approximately 200% of that of PGA-300. However, when the integrated active-quenching circuit was working at 50 ns hold-off time, the afterpulse probability distribution of PGA-384 was close to that of PGA-300. The apparent reduction of afterpulsing is attributed to the suppression of the afterpulses generated originally during the first 50 ns by active quenching. The total afterpulse probability within the range from 5 to 50 ns was only 25%, far from 200%, which indicated a much higher afterpulse probability during the first absent 5 ns. In comparison, there was no distinct difference between the afterpulse distribution with or without active quenching for PGA-300 under the condition of PDE = 8%, 1.06 μm, which indicated a much lower afterpulse probability than that of PGA-384 during the first 5 ns after detection, and hence contributed to the negligible amount of higher-order afterpulses. In general, the function of active quenching helped reduce the afterpulsing significantly for SPADs that suffer from the higher-order afterpulsing effect, such as PGA-384.

**Figure 5:**
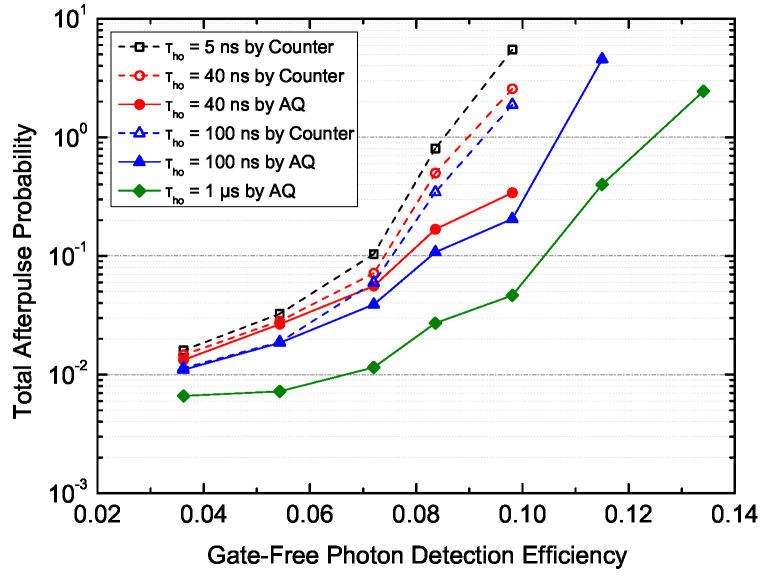
The total afterpulse probability as a function of photon detection efficiency at different hold-off times ($\tau_{\mathrm{ho}}$), 223 K, λ=1.06μm, with PGA-384. Hold-off time was applied on the SPAD by active quenching, or simply on the counter by the counter itself, as specified in the legend.

The afterpulsing performance with active-quenching-based dead time and that with counter-based dead time were compared, as shown in Figure 5. A counter-based dead time of 40 ns gave up to a 50% reduction of the total afterpulse probability compared to the 5 ns dead time, and 100 ns contributed to a 66% reduction, at a gate-free PDE of approximately 10%, 223 K for PGA-384. In comparison, with our fast active-quenching technique integrated, the SPD had afterpulse probabilities of only 6.2% and 3.7% of the 5-ns value at 40 and 100 ns dead time, respectively, or 14.3% and 31.2% of the values with counter-based dead times of 40 and 100 ns, respectively, which indicated an up to 85% reduction of the afterpulses. When the detection efficiency was relatively low, the advantage of using active quenching was not clear, e.g., a 20% reduction from the 5-ns counter-based value or a 10% reduction from the 40-ns counter-based value, at approximately 4% detection efficiency. In general, the more severe was the afterpulsing effect, the larger improvement the technique made.

In addition, similar to the gated condition, the proposed quenching technique extended the maximum gate-free photon detection efficiency at the cost of a longer dead-time, which makes the detector more flexible for practical applications.

Compared with true free-running detectors, e.g., negative-feedback avalanche diodes (NFADs), the hybrid-quenching technique exhibited a superior afterpulsing performance. Typically, NFADs have a recovery time of 100 ns, which is equivalent to a 100 ns hold-off time. The afterpulse probability of NFADs was reported as approximately 10% at 4% PDE, or 40% at 8% PDE, at 1.55 μm and 236 K [24]. Our results with PGA-300 at 1.55 μm and 223 K showed that the afterpulse probability was 4.5% at 4% PDE, and was 18.5% at 8% PDE, which are around 50% of the results of NFADs. However, the maximum gate-free detection efficiency of the proposed SPD with PGA-300 was only 9.3% at 1.55 μm, which is much lower than that of true free-running SPDs with at least 20% PDE. As this is due to the low duty cycle of the high-frequency gating, one may turn it off and use only active quenching with a longer hold-off time, such as 5 μs, if a higher PDE is desirable.

**Figure 6:**
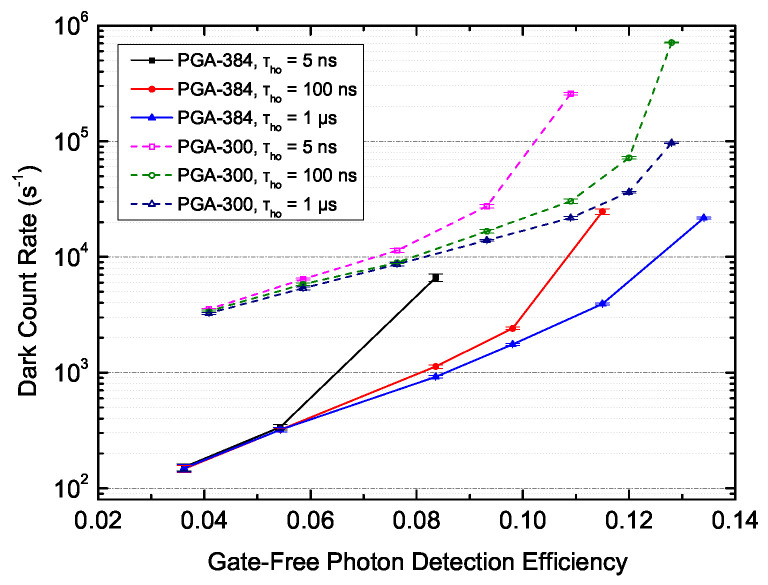
The raw dark count rate of PGA-384 and PGA-300 as a function of the gate-free photon detection efficiency at different hold-off times, 223 K, λ=1.06μm, with active quenching.

A lower afterpulse probability contributed to a significantly lower dark count rate (DCR) at high excess bias as well. The raw dark count rates of both PGA-384 and PGA-300 as a function of gate-free photon detection efficiency at different hold-off times are shown in Figure 6. Since PGA-384 has an InGaAsP absorption layer with a larger band gap than that of PGA-300 with InGaAs, it showed a generally lower DCR of 100 to 20 kHz, compared with PGA-300, which showed a DCR of 3 to 700 kHz. However, PGA-384 had a more rapid growth of the dark count rate with a higher bias and lower hold-off time, and its DCR could possibly be higher than that of PGA-300 under extreme conditions.

To our understanding, there are two reasons for this phenomenon. On the one hand, clearly a large portion of dark counts were attributed to dark-count-induced afterpulses. PGA-384, which has a larger detection area, had a higher afterpulse probability at high excess bias and a low hold-off time, and consequently the afterpulse-induced dark counts easily went out of control under such conditions. On the other hand, trap-assisted tunneling (TAT) is considered to be responsible for another large portion of dark counts, which becomes severe rapidly with excess bias. PGA-384 has a larger InP multiplication layer and accordingly more traps, which gives rise not only to afterpulses but also to TAT-induced dark counts.

Compared with our free-running SPDs with exactly the same SPAD device [25], high-frequency gated SPDs had relatively higher DCRs at similar detection efficiencies and temperatures under gate-free conditions. The difference in the DCR should also be attributed to TAT. On the one hand, the dark counts induced by thermally generated carriers in the absorption layer share similar avalanche mechanisms with photon detection, and hence are considered similar in number for both detectors at the same detection efficiency and temperature. On the other hand, dark counts induced by TAT in the InP multiplication region are regarded as the dominant source of dark counts at high excess bias voltage [24]. For high-frequency gated detectors, though the average detection efficiency was relatively low, the excess bias for photon detection was as high as 3 to 7 V, while free-running detectors required only approximately 1.5 to 2.5 V to achieve the same detection efficiency range of 6% to 13% [25]. Nevertheless, the afterpulses, at minimum, were suppressed to achieve a relatively lower DCR with the help of active quenching.

**Figure 7:**
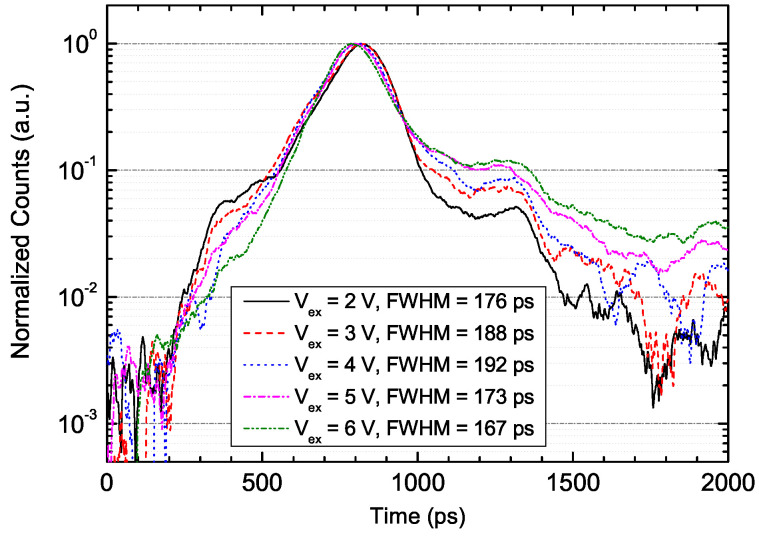
The timing jitter of PGA-300 at different excess biases, 223 K, λ=1.06μm.

Another merit of using the proposed hybrid-quenching technique in free-running applications is a low and relatively fixed timing jitter, compared with true free-running detectors with passive-quenching or active-quenching techniques. In those detectors, low timing jitter requires high excess bias, e.g., 5 V for 172 ps [25], along with high afterpulse probability, in particular at low dead times. The timing jitter of PGA-300 is shown in Figure 7. The jitter FWHM varied slightly from 167 to 192 ps within the range of 2 to 6 V. This is useful when one wants to achieve a low dead time, low afterpulse probability, and low jitter in free-running mode at the same time, with a reasonable detection efficiency of 10%, for example. The time of the detection peak varied only by tens of picoseconds, and this indicates that the delay was not related to the excess bias. This is likely beneficial in applications, such as LiDAR, where an adaptive detection efficiency is required and a change in the timing delay or jitter is less acceptable.

## 5. Conclusions

In this paper, a hybrid quenching method was presented to combine the advantages of both active quenching and high-frequency gated-passive quenching. By using a high-bandwidth RF transformer with a center tap and high-speed signal conditioning circuits, the excess bias of the SPAD could be quickly lowered for an adjustable hold-off time after a detection. Our results demonstrated that the afterpulses were successfully suppressed within the first tens of nanoseconds of true hold-off time by hybrid quenching for SPAD PGA-384, and the following higher-order afterpulses were prevented. As a result, this contributed to a significantly lower afterpulse probability and dark count rate compared with the counter-disabling method. The maximum detection efficiency was also improved due to the suppression of false counts at high excess bias voltage. Besides, as the peak excess bias required for high-frequency gating to reach a certain gate-free detection efficiency was much higher than that of the free-running condition, the proposed method demonstrated superior jitter performance to those of InP-based free-running SPDs at similar detection efficiencies.

However, there are also drawbacks to this method. The method was not equally effective for use with PGA-300, which intrinsically has a lower afterpulse probability than that of PGA-384. The dark count rate rose faster with excess bias, and was essentially higher than that of the free-running condition because of the high peak excess bias required for the method.

In addition, the proposed method can also be applied on not only PGA-384 and PGA-300 but also other high-frequency gated detectors with different application scopes for lower afterpulsing at high detection efficiencies, or for a higher maximum detection efficiency.
